# Changes in the Interaction Properties of Antibodies with Fc Receptors upon Binding to Target Antigens

**DOI:** 10.3390/bios15110759

**Published:** 2025-11-14

**Authors:** Artem S. Grevtsev, Anton A. Kommer, Irina S. Zelmanchuk, Andrei S. Avdiushkin, Elizaveta O. Ermolaeva, Aleksandr A. Tiulin, Darya O. Chernyshova, Alexandra D. Azarian, Alexandr A. Gordeev, Aleksey K. Misorin

**Affiliations:** Joint Stock Company BIOCAD, 198515 Saint Petersburg, Russia; kommer@biocad.ru (A.A.K.); zelmanchuk@biocad.ru (I.S.Z.); avdiushkinas@biocad.ru (A.S.A.); ermolaevaeo@biocad.ru (E.O.E.); tiulin@biocad.ru (A.A.T.); chernyshovado@biocad.ru (D.O.C.); azarian@biocad.ru (A.D.A.); gordeevaa@biocad.ru (A.A.G.); misorin@biocad.ru (A.K.M.)

**Keywords:** Fc receptors, FcRn, antibody, biolayer interferometry

## Abstract

The interaction of therapeutic antibodies with Fc receptors is an important property that is actively modified to improve pharmacokinetic profiles and optimize antibody-dependent mechanisms of action. Various modifications of the Fc and hinge regions of antibodies, leading to a change in affinity with various Fc receptors, are widely covered in the literature. However, data on changes in antibody and Fc receptor interactions after antibody binding to the target antigen are poorly covered in the literature. In this work, we demonstrated a change in the affinity of the interaction of antibodies with Fc receptors after binding to the target antigen via the method of biolayer interferometry. An interesting result was a significant weakening of the interaction of FcRn and FcγRIIIa with some of the antibodies when the latter bound to the target antigen, which suggests the importance of this effect for the pharmacokinetic properties and effector mechanisms of action necessary in the treatment of oncological diseases. The sensor-based biolayer interferometry methods presented in this paper allow antibody screening to be performed to detect the effects of the reduced affinity of interactions with Fc receptors, and can be a useful tool in the early development of therapeutic antibodies.

## 1. Introduction

Therapeutic monoclonal antibodies and antibody-like recombinant proteins are widely used in the treatment of oncological and autoimmune diseases. The development of modern therapeutic antibodies includes the process of optimizing specific binding to the target antigen and the effector properties of the molecule. The specificity of therapeutic molecules to target antigens is usually achieved by selection methods and can be further optimized by introducing mutations into the antigen-binding fragment of the molecule. Specificity can also be optimized by creating multivalent molecules by combining several antigen binding fragments into a single molecule. More detailed information on the various forms of antibodies, antibody fragments, and antibody-like proteins can be found in the reviews [1,2]. The effector functions of antibodies and other important properties, such as recycling and internalization, are mediated through the interaction of Fc-receptors and some soluble plasma proteins with the Fc fragment of the antibody. There are five classes of antibodies, IgM, IgD, IgG, IgA, and IgE, and their corresponding receptors, FcμR, FcδR, FcγR, FcaR, and FCϵr. More information about the classes and subclasses of antibodies, receptors, and mechanisms of signaling pathways’ activation and inhibition through their interaction with Fc receptors can be found in the articles [3,4]. Among all the classes, therapeutic molecules are mainly created based on IgG. In this regard, in the process of designing therapeutic antibodies and antibody-like proteins, interactions with Fc gamma receptors [5,6,7,8,9,10,11,12], complement system proteins (C1q) [5,13], and the neonatal Fc receptor (FcRn) are optimized [12]. Interaction with Fc gamma receptors provides effector properties such as antibody-dependent cytotoxicity and phagocytosis [14], since the corresponding receptors are present on phagocytes [15], NK cells [16], T lymphocytes [17], and many other types of cells of the human immune system involved in the formation of an immune response [18]. Interaction with complement proteins provide complement-dependent cytotoxicity [5]. Interaction with FcRn allows internalization of antibodies bound to the antigen into endosomes, followed by breakage of antibody–antigen interactions and disposition of the antigen, while returning the antibodies back to the bloodstream [19]. Thus, FcRn participates in the antibody recycling process and prolongs the antibody half-life. The optimization of the properties of antibodies binding with Fc receptors to improve the pharmacokinetics and effectiveness of therapeutic drugs, as well as examples of FDA-approved drugs with various modifications of the Fc fragment, are presented in [20,21,22].

During circulation, antibodies bind to both target antigens and various Fc receptors to perform their physiological functions. It is usually assumed that these processes are independent or at least do not influence each other significantly due to the presence of a mobile and flexible hinge region in antibodies. However, there is a number of papers where authors describe the possible existence of allosteric effects of mutual influence between antigen-binding (variable) domains and sufficiently distant domains of the Fc part of the antibody [23,24,25,26].

**Scheme 1:**
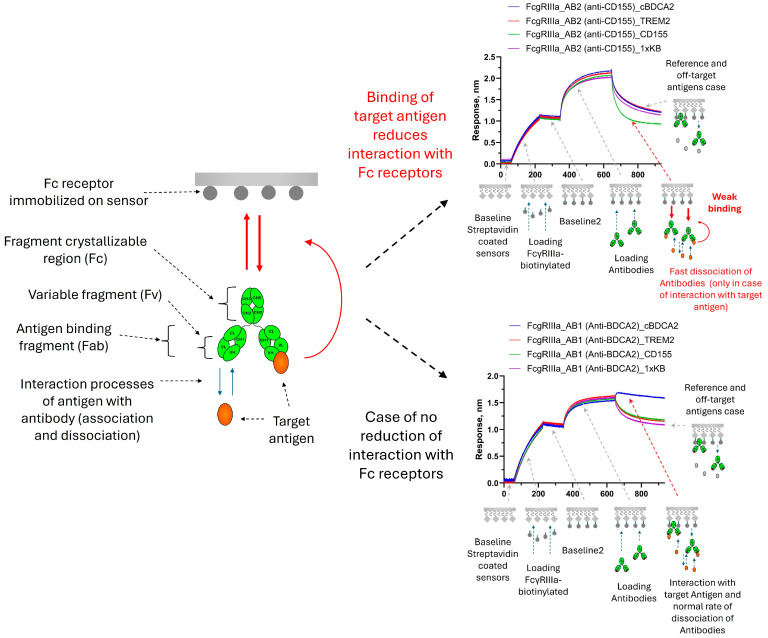
Schematic representation of the antibody structure and modification of the interaction properties with Fc receptors after the binding of the target antigen; the sensorgrams on the right side of the scheme show an example of BLI measurements for FcγRIIIa receptors.

Among the methods that can detect conformational changes, there are relatively simple approaches such as surface plasmon resonance (SPR) and biolayer interferometry (BLI). They make it possible to identify binding with high accuracy and investigate changes in the kinetics of protein interaction. These sensor methods allow us to work with proteins in a wide range of affinities, including applications for studying the kinetics of interactions between antibodies and Fc receptors. Our attention was drawn to an article [27] that demonstrated a relatively simple approach to detecting changes in the interaction of protein A and G with antibodies after binding to the target antigen. It is of little interest from the point of view of therapeutic antibody properties; however, it suggests the possibility of detecting similar effects with Fc receptors by sensor methods. In article [26], the authors mentioned that they did not detect binding by the SPR method for modified antibodies with LALA mutations (L to A mutations at residues 234 and 235 in the lower hinge region). However, for effector antibodies, the SPR and BLI methods should be sufficiently sensitive to changes in affinity for Fc receptors. In article [28], the authors demonstrated the ability to detect changes in affinity to Fc receptors after antigen binding using the SPR and BLI methods. The authors presented significant differences in the results obtained by SPR and BLI, and a lack of correlation with the isothermal titration calorimetry (ITC) results. We consider it quite acceptable to have differences between SPR and BLI, due to the difference in chip surface and sensitivity. Currently, there is no clear picture in the literature regarding the analysis of kinetic data in systems with antigen, antibody, and Fc receptors. We believe that additional data will help to better formulate the applicability limits of different assay methods. In this paper, we describe a screening approach, using BLI, for detecting the influence of antibody interaction with the target antigen on the interaction with Fc receptors. (Scheme 1). We chose BLI due to its lower protein consumption. For recording the long-term stages of protein association, flow cells on typical SPR analyzers result in a higher consumption compared to the BLI plate format. We believe that all the experiments described can be easily adapted to the SPR if necessary.

## 2. Materials and Methods

### 2.1. Expression and Purification of Antibodies and Antigens

Blood dendritic cell antigen 2 of macaca fascicularis (cBDCA-2) extracellular fragment (45–213 aa, NCBI Reference Sequence XP_005570080.1) was obtained with His-tag and C-tag at the C-terminus (C-tag consisted of four amino acids EPEA). Nectin-like protein 5 (NECL-5 or CD155) extracellular fragment (28–343 aa, NCBI Reference Sequence NP_001129242.2) was obtained with His-tag and C-tag at the C-terminus. Triggering receptor expressed on myeloid cells 2 (TREM-2) extracellular fragment (19–174 aa, NCBI Reference Sequence NP_061838.1) was obtained with His-tag, Avi-tag, and C-tag at the C-terminus. Soluble neonatal Fc receptor (FcRn) was produced as β2-microglobulin (2–120 aa, NCBI Reference Sequence NP_004039.1) at the C-terminus fused to glycine–serine linker and the IgG receptor FcRn large subunit p51 (25–297 aa, NCBI Reference Sequence NP_001129491.1), with Avi-tag and C-tag at the C-terminus. Receptor FcγRIa (16–292 aa, NCBI Reference Sequence NP_000557.1) was obtained with Avi-tag and C-tag at the C-terminus. Receptor FcγRIIIa-176V (18–208 aa, NCBI Reference Sequence NP_000560.7 with replacement of F with V in position 176) was obtained with Avi-tag and C-tag at the C-terminus.

All recombinant proteins were expressed in a Chinese hamster ovary (CHO) (BIOCAD, Saint Petersburg, Russia) cell line through transient transfection with polyethyleneimine (PEI). NECL-5, cBDCA-2, and TREM-2 were purified on immobilized metal affinity chromatography using Ni-NTA Superflow (QIAGEN GmbH, Hilden, Germany) resin, followed by size exclusion chromatography on a HiLoad 16/600 Superdex 75 pg column (Cytiva, Uppsala, Sweden). Purity was assessed by sodium dodecyl sulfate polyacrylamide gel electrophoresis (SDS-PAGE) under reducing conditions and was greater than 85% for all antigens.

The recombinant extracellular domains of Fc receptors were biotinylated by co-expression with the biotin ligase BirA during cultivation. Fc receptors were purified by affinity chromatography using Capture Select C-tag XL (Thermo Scientific, Leiden, The Netherlands) resin specific to C-tag. Additional purification by exclusive chromatography on a HiLoad 16/600 Superdex 200 pg (Cytiva, Uppsala, Sweden) column was used for FcγRIa. After the purification samples were dialyzed into a buffer solution (20 mM Tris (AppliChem, Darmstadt, Germany)-HCl , 150 mM NaCl, pH 7.5, with the addition of 5 mM dithiothreitol) and stored at −70 °C until measurements were carried out. The degree of purity estimated by SDS-PAGE under reducing conditions was more than 90% for FcRn and FcγRIIIa, and 78% for FcγRIa.

The amino acid sequence of the AB1 antibody specific to BDCA-2 (BIIB059) consisted of variable fragments of the light (SEQ ID NO:3) and heavy (SEQ ID NO:4) chains from the US20180362652 A1 patent document and IgG1 constant region. The amino acid sequence of the AB2 antibody specific to NECL-5 (NTX1088) consisted of variable fragments of the light (SEQ ID NO:1) and heavy (SEQ ID NO:2) chains from the WO2021070181 patent document and IgG1 constant region. The C-terminal amino acids G and K were removed in the heavy chain; the light chain of the kappa type was applied. Antibodies were produced in the CHO (BIOCAD, Saint Petersburg, Russia) cell line and purified by affinity chromatography using protein A resin MonSelect (BIOCAD, Saint Petersburg, Russia). Antibodies were concentrated, transferred by dialysis into 20 mM sodium acetate buffer solution with pH 5.0, and stored at −70 °C. The purity of antibodies was controlled by size exclusion high-performance liquid chromatography (SE-HPLC) on Agilent 1100 system. Analysis was carried out using TSKgel G3000SWxl 300 mm × 7.8 mm I.D. column (Tosoh, Yamaguchi, Japan) and the signal was detected at a wavelength of 280 nm. Isocratic elution mode was used with a flow of 0.5 mL/min in a mobile phase of 100 mM Na2HPO4 and 200 mM NaCl at pH 6.9. Antibody fragments’ purities were assessed by SDS-PAGE under non-reducing conditions. The purity of the obtained antibodies was greater than 97% by SE-HPLC and greater than 98% by SDS-PAGE.

### 2.2. Biolayer Interferometry (BLI)

The biolayer interferometry measurements were carried out on the OCTET RED384 system (PALL Fortebio, Menlo Park, CA, USA). All measurements were carried out in three independent runs to verify reproducibility. All experiments were performed at 30 °C with orbital mixing at 1000 rpm, using kinetic buffer solution consisting of phosphate-buffered saline 4.3 mM Na2HPO4, 136.9 mM NaCl, 1.5 mM KH2PO4, 2.7 mM KCl (Sigma-Aldrich, St Louis, MO, USA), 0.1% (*v*/*v*) Tween 20 (PanReac, Barcelona, Spain), and 0.1% (*w*/*v*) bovine serum albumin (Sigma-Aldrich, St Louis, MO, USA) at pH 7.4. Before measurements, the sensors were equilibrated in kinetic buffer solution for at least 20 min. During the measurements, the sensors were immersed in wells with various solutions introduced into standard 384-well plates.

#### 2.2.1. Modification of the Binding Properties of Antibodies to Protein A

The modification of the binding properties of antibodies to protein A was tested using ProA sensors (Sartorius, Shanghai, China). The experiment consisted of the following: recording the baseline for 30 s, immobilization of the antibodies to sensors for 300 s, a baseline after the immobilization of antibodies for 120 s, and association for 7200 s. The antibody immobilization step was performed in a solution with a concentration of 10 micrograms/mL. At the association stage, sensors with immobilized antibodies were immersed in wells with antigen solutions with a concentration of 10 μg/mL, and in a buffer solution without antigens as a reference.

#### 2.2.2. Modification of the Binding Properties of Antibodies to c FcRn

The modification of the binding properties of antibodies to c FcRn was tested using SA sensors (Sartorius, Shanghai, China). All the steps of this experiment were carried out in a kinetic buffer with a pH of 6.0 (the pH was adjusted by adding 5M H_3_PO_4_). The experiment consisted of a baseline recording for 60 s, the immobilization of biotinylated FcRn receptors on the sensors, a baseline after the receptor immobilization for 60 s, antibody immobilization for 180 s, a baseline (30 s), and association (interaction with antigens) for 300 s. FcRn immobilization was performed in a receptor solution with a concentration of 5 μg/mL to a loading signal level of 1 nm. Antibody immobilization was performed in a solution with a concentration of 10 μg/mL. At the association stage, sensors with immobilized antibodies were immersed in wells with antigen solutions with a concentration of 20 μg/mL.

#### 2.2.3. Modification of the Binding Properties of Antibodies to c FcγRIa

The modification of the binding properties of antibodies to c FcγRIa was tested using SA sensors (Sartorius, Shanghai, China). The experiment consisted of recording a baseline for 30 s, the immobilization of biotinylated receptors on the sensors, a baseline after the immobilization of receptors for 60 s, the immobilization of antibodies for 300 s, and association (interaction with antigens) for 600 s. The FcγRIa immobilization step was carried out in a receptor solution with a concentration of 5 μg/mL and a loading signal level of 1 nm (the signal level for completing the loading stage was set as a parameter in the control software). At the association stage, sensors with immobilized antibodies were immersed in wells with antigen solutions with a concentration of 10 μg/mL.

#### 2.2.4. Modification of the Binding Properties of Antibodies to c FcγRIIIa

The modification of the binding properties of antibodies to c FcγRIIIa was tested using SA sensors (Sartorius, Shanghai, China). The experiment consisted of recording a baseline for 60 s, the immobilization of biotinylated receptors on sensors, a baseline after the immobilization of receptors for 120 s, the immobilization of antibodies for 300 s, and association (interaction with antigens) for 300 s. The FcγRIIIa immobilization step was carried out in a receptor solution with a concentration of 5 μg/mL and a loading signal level of 1 nm. At the association stage, sensors with immobilized antibodies were immersed in wells with antigen solutions with a concentration of 10 μg/mL.

#### 2.2.5. Interaction of Antigen and Antibody Complexes with FcγRIIIa

The interaction of antigen and antibody complexes with FcγRIIIa receptors was tested using SA sensors (Sartorius, Shanghai, China). The experiment consisted of recording a baseline for 60 s, the immobilization of biotinylated receptors on the sensors, a baseline after the immobilization of receptors for 120 s, and association (interaction with premixes of antibodies and antigens) for 300 s. The FcγRIIIa immobilization step was carried out in a receptor solution with a concentration of 5 μg/mL and a loading signal level of 1 nm. At the association stage, sensors with immobilized receptors were immersed in wells with solutions comprising a mixture of antibodies and antigens in a molar ratio of 1:4 (the molar ratio of Fab fragments and antigens was 1:2). The required amount of antigen was calculated for a solution with a final antibody concentration of 10 μg/mL.

In the majority of the experiments described after the association stage, there was a dissociation stage, at which the sensors were immersed in a kinetic buffer solution. For the ProA sensors, after the dissociation stage, there was an additional regeneration stage in a 10 mM solution of glycine (PanReac, Barcelona, Spain)–HCl (SIGMATEK, Khimki, Russia) pH2.0. Dissociation and regeneration in this work were recorded as technical (auxiliary) steps and were not used to interpret the results. To simplify figures, these steps were removed from the presented sensorgrams when processing the results. Various variants of non-specific interactions were tested. There were no non-specific interactions of antibodies and antigens with empty sensors, antigens with Fc receptors on sensors, or antigen storage solution with antibodies immobilized on Fc receptors (effect of antigen storge buffer, diluted identically to studied antigen solutions).

### 2.3. Enzyme-Linked Immunosorbent Assay (ELISA)

All incubation steps were carried out at 25 °C, with shaking at 300 rpm. After each incubation step, the plate was washed with five cycles of dispensing and aspiration with 300 μL per well of phosphate-buffered saline (PBS) wash solution supplemented with 0.05% (*v*/*v*) Tween 20 (PanReac, Barcelona, Spain). Biotinylated FcγRI and FcγRIII receptors were immobilized in a 96-well plate coated with streptavidin (Thermo Scientific, Rockford, IL, USA). For immobilization, receptors solutions in PBS with concentrations of 1 μg/mL and 2.5 μg/mL for FcγRI and FcγRIII, respectively, were added to the wells at 100 μL per well. The plate was incubated for 60 min and washed. PBS with 1% bovine serum albumin (Sigma-Aldrich, St Louis, MO, USA) was added to all wells of the plate and incubated for 10 min, then washed. The following samples were then added: AB1 antibodies, premix AB1 and cBDCA-2, premix AB1 and NECL-5, premix AB1 and TREM-2, AB2 antibodies, premix AB2 and cBDCA-2, premix AB2 and NECL-5, and premix AB2 and TREM-2. All premixes were prepared in a molar ratio of antibodies to antigens of 1:3, and the concentration of antibodies in the final solution of all samples was 2.5 μg/mL for FcγRIa receptors and 10 μg/mL for FcγRIIIa receptors. Each sample was measured in triplicate on a plate within a single ELISA run. Wells with immobilized receptors without antibodies were used as a reference. As negative controls, similar premixes were added to wells without immobilized Fc receptors. The samples were incubated for 60 min, after which the plate was washed. Then secondary antibodies were added at a dilution of 1:5000 and the plate was incubated for 5 min and washed (the short incubation time was chosen to reduce the number of antibodies dissociated from the plate, which is due to the low affinity of FcγRIIIa receptors for antibodies). Next, 100 µL of tetramethylbenzidine substrate (Bektor Corp., Aurora, CO, USA) was added per well. The reaction was stopped after 5 min by adding 100 µL of 1M H2SO4 (SIGMATEK, Khimki, Russia). Immediately after stopping the reaction, optical density was measured using a Sunrise microplate reader (Tecan Austria GmbH, Grödig, Austria). The microplate measurement parameters were set in the Magellan Pro software (version 7.4.0.4, Tecan Austria GmbH, Grödig, Austria), the absorption wavelength was 450 nm, and reference values were taken at a wavelength of 620 nm in the same wells and subtracted. The ELISA was performed in two independent replicates.

### 2.4. Antibody-Dependent Cellular Cytotoxicity (ADCC) Assay

The transgenic cell lines CD155/TCR Activator—CHO Recombinant Cell line (BPS Bioscience, San Diego, CA, USA)—and HEK BDCA-2 (BIOCAD, Saint Petersburg, Russia) were used as target cell lines, while Jurkat-CD16(V176)-NFAT-luc (BIOCAD, Saint Petersburg, Russia) was used as effector cell line.

Sixteen hours before the start of the analysis, 15,000 target cells per well were seeded in 96-well, white/clear, flat bottom plate (Corning, Glendale, AZ, USA) in a quantitative determination medium (QDM), which consisted of DMEM/F-12 (PanEco, Moscow, Russia), 2 mM L-glutamine (PanEco, Moscow, Russia), and 10% HI FBS low IgG (Gibco, Walthem, MA, USA). The plate was incubated at 37 °C, 5% CO_2_, in a CO_2_ incubator (Binder, Tuttlingen, Germany). Effector cells were incubated similarly to target cells, at a concentration of 5 × 10^5^ cells/mL, without selective antibiotics in the growth medium, which was as follows: RPMI-1640 (PanEco, Moscow, Russia), 2 mM L-glutamine (PanEco, Moscow, Russia), and 10% HI FBS (Cytiva, Marlborough, MA, USA). At the end of the incubation, the solution of antibodies and effector cells was added to the target cells in a ratio of 5:1 in QDM. The concentration of antibodies in the final solution varied from 1 µg/mL to 5 × 10^−5^ µg/mL (samples with a dilution factor of 3). The plate with target cells, effector cells, and antibody solution was incubated for 5 h at 37 °C, 5% CO_2_. After the end of the incubation, a solution of BioGlo reagent (Promega, Madison, WI, USA) was added to the plate in a 1:1 ratio to the contents of the wells and incubated for 8 min in the dark on a shaker, at room temperature. Luminescence was determined using an Infinite MPlex plate reader (Tecan Austria GmbH, Grödig, Austria).

### 2.5. Data Analysis

The results of measurements obtained by the biolayer interferometry method were exported from Octet Data Analysis software (version 9.0, ForteBio, Menlo Park, CA, USA) to comma separated values and then images were plotted in Prism (version 10.5.0, GraphPad Software, LLC, Boston, MA, USA). For visual clarity, the obtained sensorgrams were manually aligned along the response axis. Also, to simplify the perception of the graphic material, the last steps of the experiments (dissociation and regeneration) are not applied for analysis of sensorgrams. The results of measurements of the optical density of ELISA plates were processed in the Magellan Pro (version 7.4.0.4, Tecan, Grödig, Austria) software. The ADCC measurement results were fitted using a four-parameter curve in Prism.

## 3. Results and Discussion

### 3.1. Modification of Interaction Properties with Protein A

**Figure 1:**
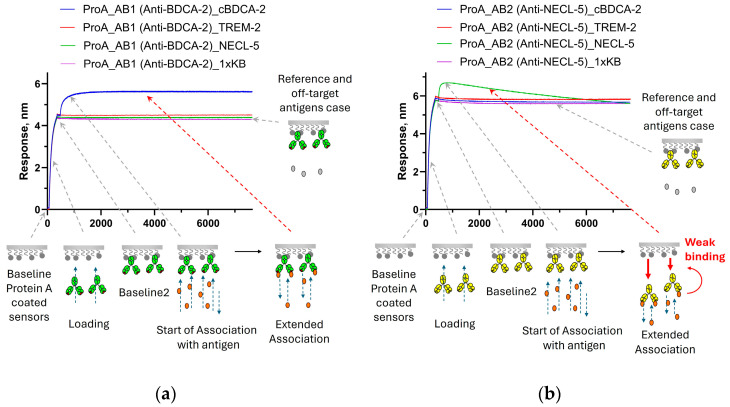
Sensorgrams of the interaction of antibodies immobilized on ProA sensors: (**a**)—binding of AB1 antibodies with the cBDCA-2 antigen occurs with the reaching of a plateau, and no interaction or effects with the TREM-2 or NECL-5 antigens were detected; and (**b**)—an example of a signal decay at the association stage for the AB2 antibody upon binding to the target antigen NECL-5, and non-target antigens cBDCA-2 and TREM-2 do not have any effect on the AB2 antibody. The stages of the experiment are described in Section 2.2.1. The effects presented on sensorgrams are discussed in the text.

**Figure A1:**
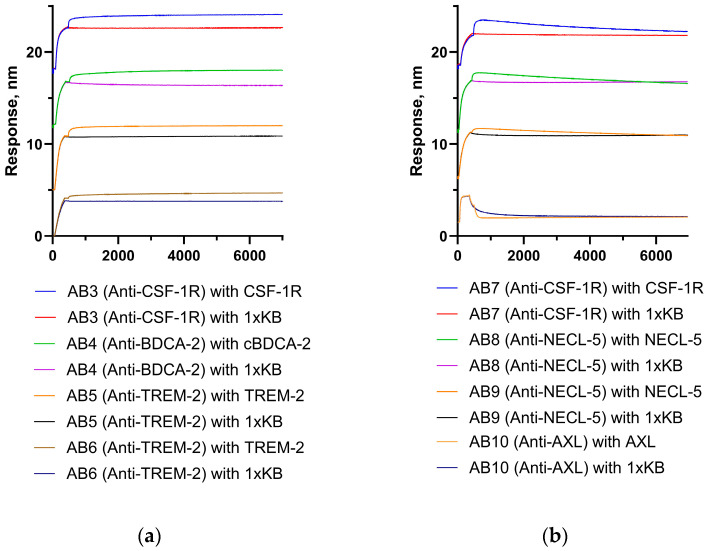
Experiment on the interaction of antibodies immobilized on ProA sensors with target antigens: (**a**)—antibodies AB3–AB6 demonstrate standard binding, and (**b**)—antibodies AB7–AB10 demonstrate a signal decay at the prolonged association stage (weakening of the interaction of antibodies with the ProA protein is observed after binding to the target antigen).

We came up with the idea of screening the properties of the Fc fragment after binding antibodies to the antigen, after repeatedly detecting unusual behavior in the antibodies during affinity screening on sensors with protein A (sensors with antibodies to the Fc fragment or Fab fragment of human IgG are also used; however, we often prefer to work with ProA due to its high resistance to regeneration cycles). Standard antigen affinity screenings were performed when loading antibodies onto ProA sensors. The sensors loaded with antibodies were then immersed in a solution with the antigen under study. The usual result of antigen binding was a non-linear increase in the signal until a plateau was reached. However, we observed that for some samples, after reaching a plateau, a rapid decrease in signal was observed (in comparison with the reference). We assumed that this was not simply an artifact of equilibration in a buffer solution containing the antigen, but a change in the binding properties of the antibody to protein A. As a result, we set up an experiment with a long recording time of the association stage for samples with such a non-standard effect when interacting with an antigen. (Figure 1). For some antibodies, the signal level drops below the values of the reference sensor right at the association stage (the reference sensor was loaded with the antibody and immersed in a buffer solution without the antigen at the association stage). The only explanation for this effect can be a weakening of the binding of the loaded antibodies to protein A after the binding of the antibodies to the antigen. It is worth noting that the interaction of non-specific antigens with the loaded antibodies (negative controls) did not lead to a decrease in the signal of non-target antibodies. The interaction of signal-decay-inducing antigens with non-target antibodies also did not lead to a decrease in the signal. Thus, it was exactly the binding to target antigens that reduced the affinity of the studied antibodies to protein A. Additional examples of antibodies that demonstrate no changes in the properties of binding to protein A, and antibodies that dissociate from protein A due to the action of target antigens are shown in Figure A1.

**Figure 2:**
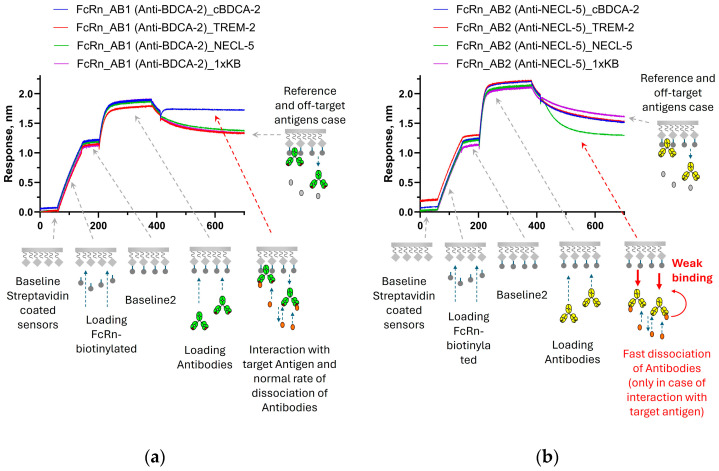
Sensorgrams of sequential steps of FcRn immobilization, antibodies’ immobilization, and association of antigens with immobilized antibodies in a kinetic buffer solution with pH 6.0: (**a**)—standard binding pattern with signal growth during interaction of AB1 antibodies with the target antigen cBDCA-2; and (**b**)—example of signal decay at the association stage for AB2 antibodies during interaction with the target antigen NECL-5 (non-target antigens do not demonstrate significant differences from the reference). The stages of the experiment are described in Section 2.2.2. The effects presented on sensorgrams are discussed in the text.

The obtained results demonstrate that the non-covalent loading of antibodies onto sensors can sometimes lead to the distortion of binding curves, which should be considered when studying kinetics. Working with low concentrations of the antigen and a short association time, this effect is practically undetectable, and, if necessary, it is always possible to check the results of kinetic measurements on the sensors with covalent loading. From our point of view, the possibility of detecting a relatively long-range effect is of much greater interest. The explanation that seems most likely to us is a conformational change in the antibody molecule. Since the CH2-CH3 binding interface is already shielded by the protein A molecule, and the antigen does not cause the dissociation of non-specific antibodies from the ProA sensors, we assume that the antigen does not act directly on protein A but changes the conformational state of the antibody. The FcRn receptor binds (at pH 6.0) to the same region of antibodies in the CH2-CH3 interface as protein A, and a similar effect was observed for this receptor with AB1 and AB2 antibodies (Figure 2). Antibody AB1 showed a standard signal increase upon the binding of the target antigen (cBDCA) to FcRn-immobilized antibodies. AB2 showed accelerated dissociation from FcRn upon binding to the target antigen. None of the antibodies altered their dissociation rates in response to non-target antigens. A correlation between the observations of the effect for protein A and FcRn can be assumed; however, such a hypothesis requires further testing on a larger number of different antibodies, which is beyond the scope of this work.

Since such a pronounced effect of presumably conformational action was observed at a site relatively far from the Fab fragment (ProA and FcRn bind at the CH2-CH3 interface of the IgG1 antibody heavy chain domains), we assumed that conformational changes are possible in the hinge region, and, therefore, the properties of the interaction with Fc gamma receptors can undergo changes as well. We decided to test the existence of the effect on the FcγRIa and FcγRIIIa receptors.

### 3.2. Modification of Interaction Properties with Fc Gamma Receptors After Binding to the Target Antigen

**Figure 3:**
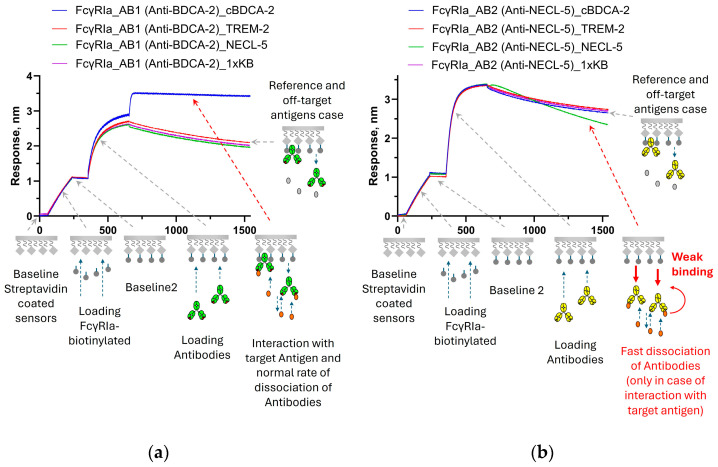
Sensorgrams of sequential steps of FcγRIa immobilization, antibodies’ immobilization, and association of antigens with immobilized antibodies: (**a**)—binding with signal growth during interaction of AB1 antibodies with antigen cBDCA-2; and (**b**)—example of signal decay at the association stage for AB2 antibodies during interaction with NECL-5. The stages of the experiment are described in Section 2.2.3. The effects presented on sensorgrams are discussed in the text.

Figure 3 shows an example of an experiment to test the change in the binding properties to FcγRIa as a result of the action of target antigens. FcγRIa was loaded onto the SA sensors, and then the antibodies were immobilized onto the receptors. In the next step, the sensors were immersed in antigen solutions and in a kinetic buffer solution without antigens (reference).

For the AB1 antibody specific to BDCA-2, there was no effect of the attenuation of the interaction with FcγRIa; the sensorgrams showed a typical increase in the signal upon binding to the target antigen. In the case of the AB2 antibody, when the sensors with FcγRIa were immersed in a solution with antibodies and then in a solution with the target NECL-5 antigen, an increase in the signal was observed within 30–50 s (Figure 3b); however, with prolonged recording of the association, the effect of dissociation from the sensors began to prevail. The obtained results indicate that the effect of the binding to the antigen in the case of FcγRIa is realized significantly slower compared to the rate of the binding of the antigen to the Fab fragment of antibodies. The signal drop below the reference for AB2 indicates a weakening of the interaction of the FcγRIa receptor with the antibody after binding of the antibody to the target antigen (NECL-5). Non-target antigens did not change the rate of the dissociation of the antibodies from the receptors, so it was exactly the binding to the target antigen that modified the interaction of antibodies with receptors.

**Figure 4:**
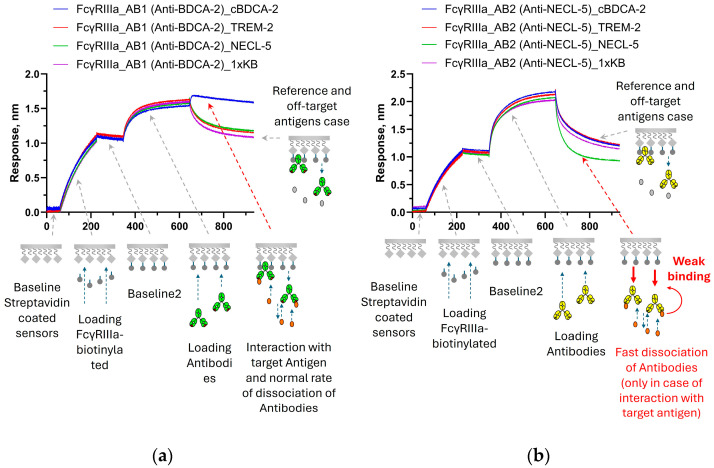
Sensorgrams of sequential steps of FcγRIIIa immobilization, antibodies’ immobilization, and association of antigens with immobilized antibodies: (**a**)—binding with signal growth during interaction of AB1 antibodies with antigen cBDCA-2; and (**b**)—example of signal decay at the association stage for AB2 antibodies during interaction with NECL-5. The stages of the experiment are described in Section 2.2.4. The effects presented on sensorgrams are discussed in the text.

Figure 4 shows the results of a similar experiment to test the modification of the properties of the interaction of antibodies with FcγRIIIa receptors. When immersed in a solution with a specific antigen, there was an increase in the signal for the BDCA-2-specific antibody AB1 compared to the reference (Figure 4a) and a decrease in the signal was observed when AB2 interacted with NECL-5 (Figure 4b). Thus, after binding to the target antigen, the affinity of AB2 antibodies to FcγRIIIa was significantly reduced.

**Figure 5:**
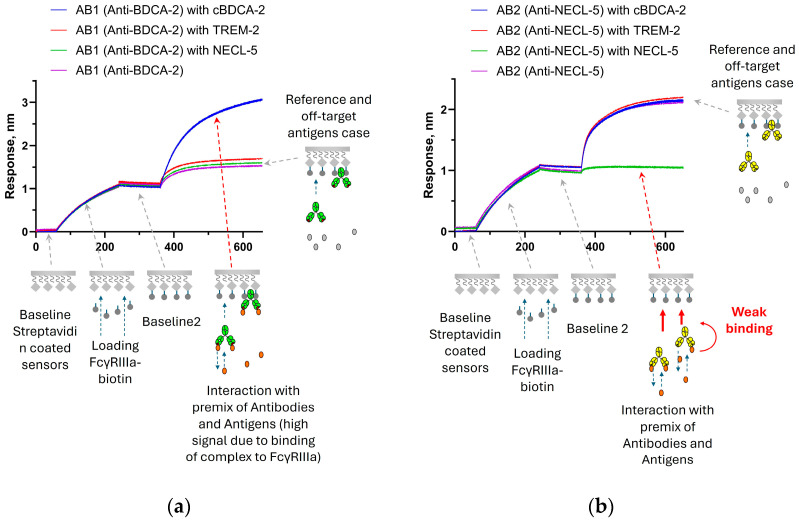
Sensorgrams of sequential steps of FcγRIIIa immobilization and interaction of antibody and antigen premixes with immobilized receptors: (**a**)—the signal for the AB1 antibody premix with the cBDCA-2 antigen is higher than the binding signal of AB1 antibodies without antigen (the signal is higher due to the attachment of AB1 and antigen complexes); and (**b**)—the binding signal of the AB2 premix with NECL-5 is significantly lower than the binding signal of AB2 antibodies and AB2 premixes with non-target antigens. The stages of the experiment are described in Section 2.2.5. The effects presented on sensorgrams are discussed in the text.

Then we decided to test whether differences between antibodies bound to the antigen (antibody–antigen premix) and antibodies without antigen could be detected. To do this, sensors with immobilized FcγRIIIa receptors were immersed in antibodies and in the antibody–antigen premix solutions, respectively. In the case of the same affinity of Fc receptors to antibodies and antibody–antigen complexes, the premix signal should exceed the antibody signal (since the antibody–antigen complexes have a higher molecular weight). This is the situation observed for the AB1 antibody (Figure 5a). However, for the AB2 antibody, the premix signal growth was lower (there is almost no binding compared to premixes with non-targeted antigens), which is in good agreement with a decrease in affinity to the Fc receptor. (Figure 5b). For AB2, even with a higher molecular weight of the antigen–antibody complex, the signal grew slower than for AB2 mixed with non-target antigens. This experiment shows that the effect of the weakening of the interaction with FcγRIIIa is also observed when the binding order changes, first with the antigen, and then with FcγRIIIa.

**Figure 6:**
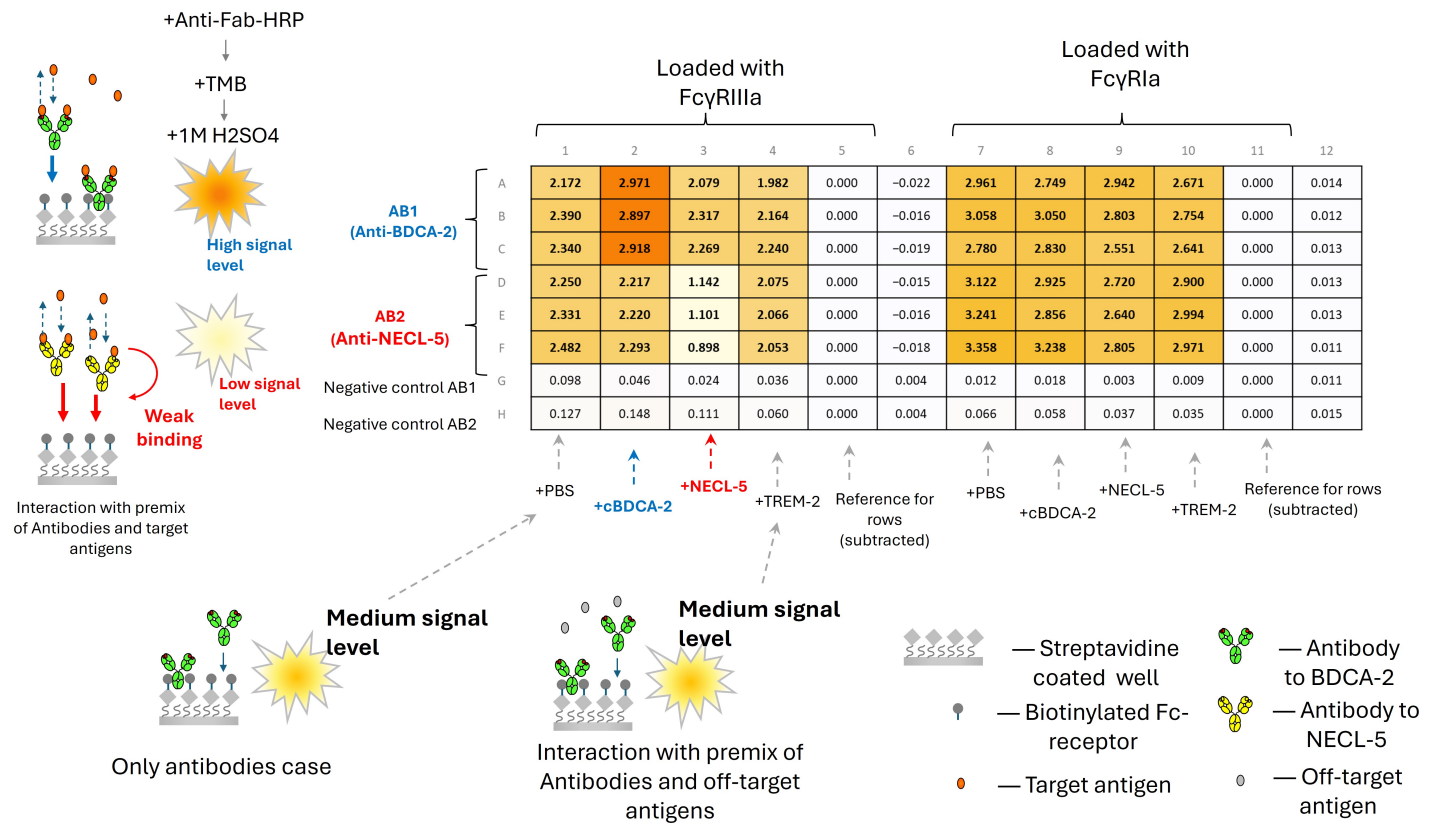
Schematic representation of the test of the interaction of immobilized receptors FcγRIIIa and FcγRIa with premixes of AB1 antibodies and antigens, cBDCA-2, NECL-5, and TREM-2, and AB2 antibodies and antigens cBDCA-2, NECL-5, and TREM-2 by the ELISA method.

We also decided to apply the ELISA method due to its simplicity. Since the most pronounced effect of the weakening of the interaction of antibodies with Fc receptors was observed in the experiment with the premixes of antigen and antibodies, we tried a similar measurement scheme (immobilization of receptors and checking binding with premixes). The obtained effect of changing the binding properties of antibodies to FcγRIIIa was detected by the ELISA method (Figure 6).

When premixes of the antibody with the target antigen were added, a decrease in the intensity of binding to immobilized FcγRIIIa receptors by approximately two times (according to the change in detectable optical density) was observed compared to the premixes of non-target antigens for the AB2 antibody. At the same time, the opposite effect was observed for the AB1 antibodies (an increase in the signal intensity by approximately 1.5 times), which is consistent with the results obtained by biolayer interferometry methods. We were unable to detect differences between the premixes with different antigens for AB1 and AB2 to the FcγRIa receptor using the ELISA method, which may be due to requirement of ELISA parameters optimization for this experiment. However, the absence of an effect of the attenuation of the FcγRIa binding signal for the AB2-NECL-5 complex allows us to assume that there is no steric blocking of secondary antibody binding sites by the antigen. Thus, we assume that the antigen bound to the target antibody has a negligible effect on the intensity of staining with secondary antibodies. However, we believe that the ELISA method requires more detailed testing on a larger number of samples and various secondary antibodies to exclude hypotheses about the influence of secondary antibodies on the obtained result. Also, during staining with secondary antibodies, dissociation of primary antibodies occurs, which leads to a decrease in sensitivity after washing the plate from secondary antibodies. The approach, based on biolayer interferometry, does not require additional secondary antibodies and allows us to immediately detect the binding signal, which turned out to be convenient when studying the properties of Fc receptors.

**Figure A4:**
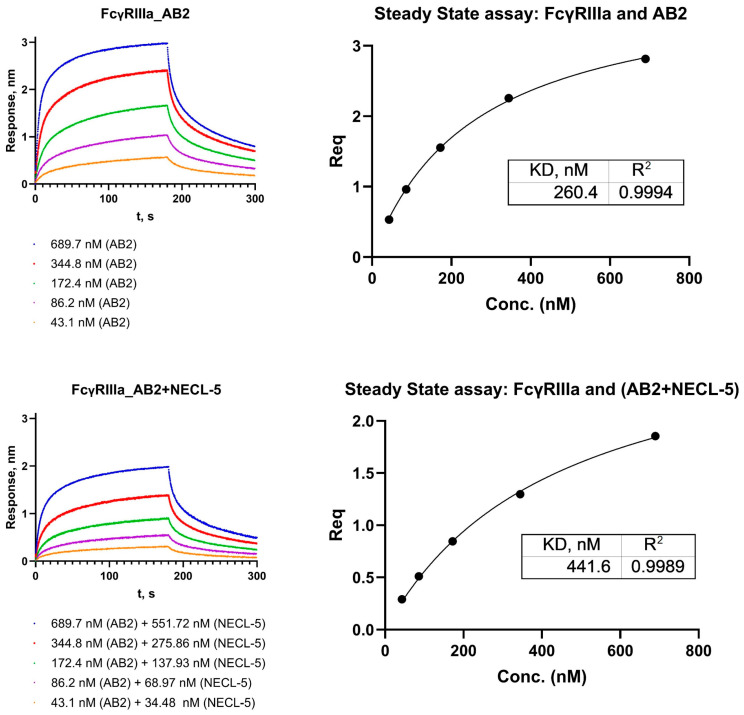
Analysis of the kinetics of interaction of FcγRIIIa receptors with AB2 antibodies and complexes AB2 with NECL-5.

In this paper, we used the interpretation of the biolayer interferometry results in the format of whether effect exists or not on sensorgrams to simplify the presentation of the results. For the cases presented in Figure 1, Figure 2, Figure 3 and Figure 4, the empirical Equation (A1) (an empirical modification of the 1:1 interaction equation) given in Appendix A can be used. In our case, such an empirical equation, despite several significant simplifications, described the association stage of interest quite well for the experiments with all Fc receptors and ProA sensors. However, it is worth noting that the kinetic constants obtained, as a result of the approximation in such empirical models with a large number of parameters, are suitable only for comparative analysis. Different approximation procedures with different initial conditions can give different sets of selected parameters. The process of adjusting the approximation requires considerable time and the selection of initial parameters based on the analysis of the obtained sensorgrams and orthogonal measurement data. An easier way to numerically estimate the observed effect can be carried out based on the analysis of equilibrium states [27], using the measurement scheme shown in Figure 5. The approach proposed in [27] usually requires obtaining five to six sensorgrams for different antibody concentrations, after which the data are reduced to linear regression (Scatchard or Sips equations) or approximated using Equation (A5) to obtain numerical values of the equilibrium dissociation constant KD (the authors [27] used the equilibrium constant of the association Ka, which is equivalent to 1/KD). We have given an example of using the steady-state data processing method to analyze the kinetics of FcγRIIIa interaction with AB2 antibodies, and complexes AB2 with NECL-5 (Figure A4). The obtained value of the KD constant in three measurements for the AB2 antibody to FcγRIIIa was (259.2 ± 1.1) nM, and the KD constant for the AB2-NECL-5 to FcγRIIIa complex was (493.4 ± 46.5) nM (data is presented in the format average ± standard deviation). The small value of the standard deviation in the case of AB2 with FcγRIIIa is likely accidental. It is worth noting that the analysis of equilibrium states is also only an approximation of the real, studied system of the interaction of receptors with a premix of antibodies and antigens. We recommend using the values of KD (or Ka) obtained by the steady-state method for comparative analysis within the framework of one fixed measurement method. In our opinion, for screening comparison it could be sufficient to determine the signal level (response) achieved when the curves reach a plateau (or at the end of the association stage).

**Figure 7:**
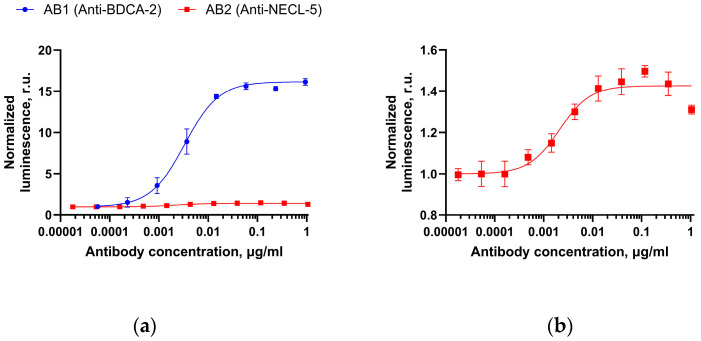
Antibody-dependent cellular cytotoxicity (ADCC) assay with Jurkat-CD16(V176)-NFAT-luc effector cells: (**a**)—transgenic cell line with BDCA-2 expression for the AB1 effector antibody (Anti-BDCA-2) is shown by the blue line and the target CD155/TCR Activator–CHO Recombinant Cell line (BPS Bioscience) for the AB2 antibody (Anti-NECL-5) is shown by the red line; and (**b**)—enlarged image of the ADCC test results for the AB2 antibody. Blue and red error bars represent standard deviations of normalized luminescence (for three repetitions) for AB1 and AB2, respectively.

Based on the results obtained by the BLI method, low ADCC efficiency should be expected for the AB2 antibody. The results of ADCC assay on the Jurkat-CD16(V176)-NFAT-luc reporter line are shown in Figure 7.

A low ratio of the upper to lower plateau signal of the four-parameter curve was observed for the AB2 antibody. The obtained ratio (less than two) is not typical for effector antibodies in this assay, indicating the reduced ADCC (usually the upper to lower plateau ratio is above five). A typical dependence of luminescence on the concentration of an effector antibody with similar Fc fragment is shown in Figure 7, using the example of the AB1 antibody (Anti-BDCA-2). The results for the AB1 (Anti-BDCA-2) antibody were applied only as a rough comparison sample, demonstrating typical upper and lower plateau ratios in reporter assays with Jurkat-CD16(V176)-NFAT-luc effectors. Further testing on a larger number of target cells for different antibodies demonstrating the described effect is necessary to confirm the effector properties weakening upon binding to the target antigen more rigorously.

Strictly speaking, our results only indirectly indicate the possibility of a conformational mechanism for changing the properties of the Fc fragment after the interaction of antibodies with the target antigen. However, the steric shielding of the binding site alone (without conformational changes in the Fc fragment) does not explain the acceleration of dissociation when a target antigen interacts with an antibody already bound to the receptor (cases shown in Figure 2, Figure 3 and Figure 4). We do not exclude the possibility of steric blocking effects; however, the conformational changes in the Fc region of the antibody caused by the binding of the Fab fragment to the antigen explain all experimental observations presented in this work well. The effect described could also be considered when studying the kinetics of antibodies interaction with Fc receptors using sensors-based methods and when comparing the affinity of receptors not only with the antibodies themselves but also with antigen–antibody complexes. This paper presents results obtained for two model antibodies, so further verification of these effects on larger sets of antibodies using additional orthogonal assays is required.

## 4. Conclusions

In this work, we have demonstrated the possibility of detecting changes in the properties of binding of antibodies to Fc receptors when interacting with a target antigen, using the method of biolayer interferometry. We have shown that for some antibodies, it is possible the reduce affinity to FcRn and Fc gamma receptors (FcγRIIIa and FcγRIa) after target antigen binding. In our opinion, this effect could be caused by conformational changes in the antibody during the interaction with the target antigen. The presented methods of analysis can be applied in the process of selection and optimization of antibody candidates at the early stages of research and development of therapeutic drugs.

## Figures and Tables

**Figure A2:**
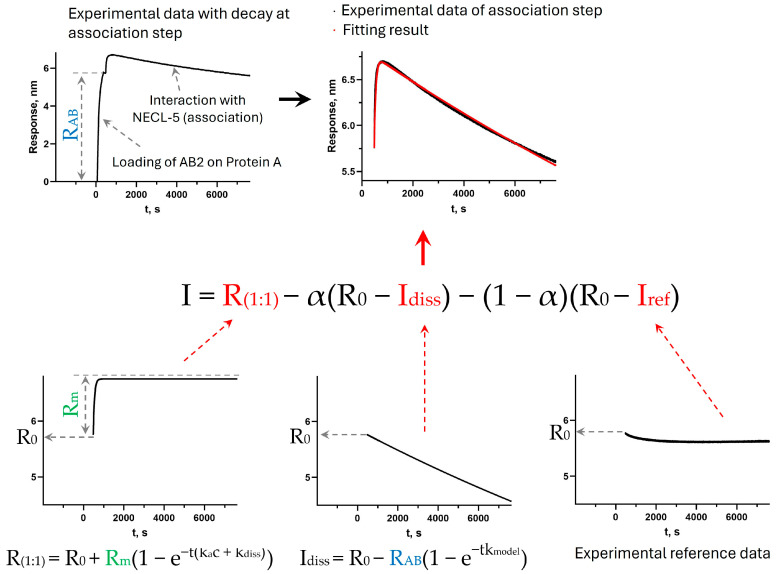
Visualization of model parameters and fitting process (example for interaction of AB2 immobilized on protein A with NECL-5).

**Figure A3:**
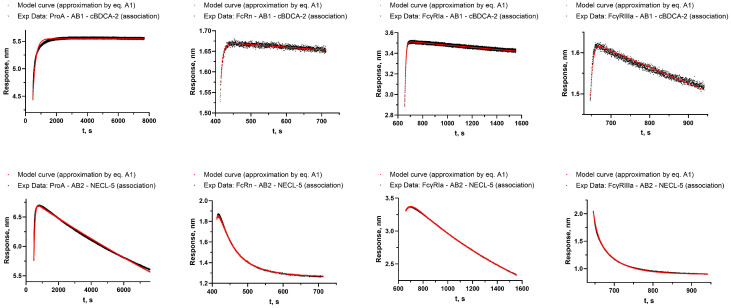
The results of the approximation according to the model described by Equation (A1) of association stage of the target antigen with antibodies AB1 and AB2 immobilized on protein A, FcRn, FcγRI, and FcγRIIIa (all sequential steps of these sensorgrams are shown in Figure 1, Figure 2, Figure 3 and Figure 4).

**Figure A5:**
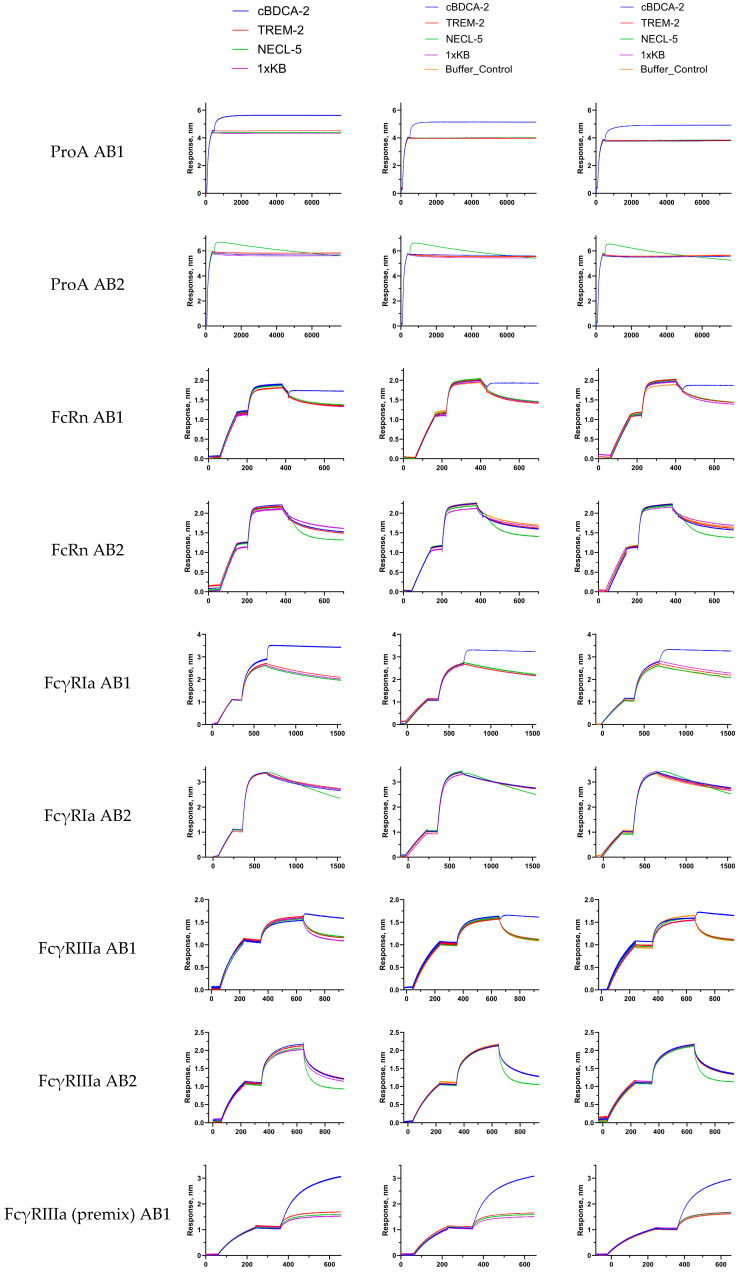

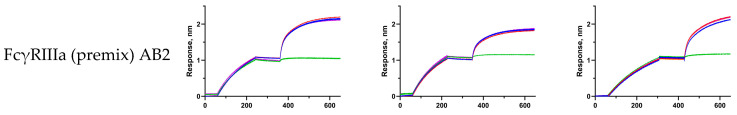
Sensorgrams (three replications) of the experiments described in Section 2.2.1, Section 2.2.2, Section 2.2.3, Section 2.2.4 and Section 2.2.5; obtained sensorgrams were manually aligned for visual clarity.

**Figure A6:**
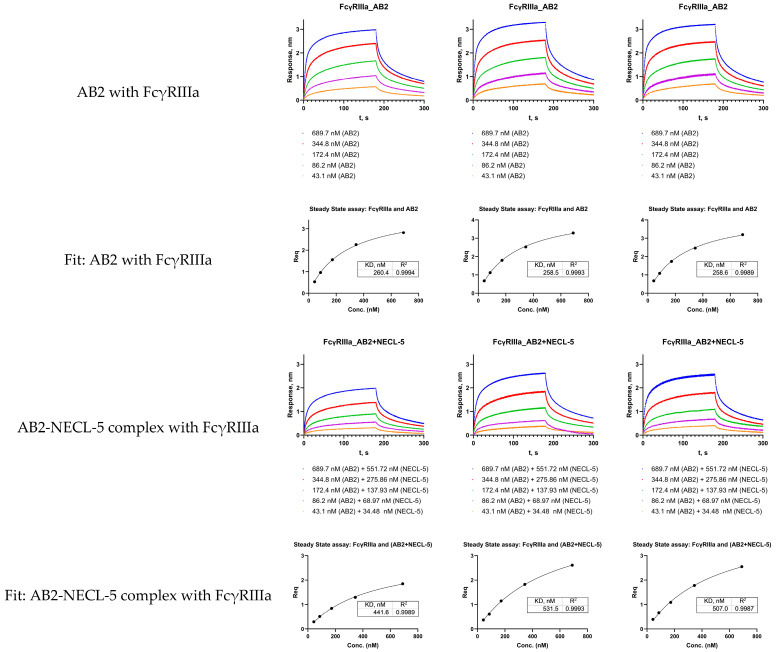
Steady-state assay for sensorgrams (three replications) of interaction AB2 with FcγRIIIa and AB2-NECL-5 complex with FcγRIIIa.

## Data Availability

The original contributions presented in this study are included in the article. Further inquiries can be directed to the corresponding author.

## Appendix A

The antigens used for measurements were extracellular fragments of the corresponding proteins. Blood dendritic cell antigen 2 of macaca fascicularis (cBDCA-2) extracellular domain (45–213 aa, NCBI Reference Sequence XP_005570080.1) was obtained with His-tag and C-tag at the C-terminus. Nectin-like protein 5 (NECL-5 or CD155) extracellular domain (28–343 aa, NP_001129242.2) was obtained with His-tag and C-tag at the C-terminus. Triggering receptor expressed on myeloid cells 2 (TREM-2) extracellular domain (19–174 aa, NP_061838.1) was obtained with His-tag, Avi-tag, and C-tag at the C-terminus. Macrophage colony-stimulating factor 1 receptor (CSF1R) extracellular domain (21–502 aa, NP_001275634.1) was obtained with Avi-tag, Flag-tag, and His-tag at the C-terminus. Tyrosine-protein kinase receptor UFO isoform 1 precursor (AXL oncogene) extracellular domain (26–446 aa of NP_068713.2) was obtained with His-tag and C-tag at the C-terminus.

The amino acid sequences of the variable fragments for the AB3–AB10 antibodies were obtained by standard immunization and selection methods using phage display. Antibodies AB3 and AB7 are specific to CSF1R, AB4 to BDCA 2 antigen, AB5 and AB6 to TREM 2 antigen, AB8 and AB9 are specific to CD 155, and AB10 is specific to AXL. The AB3–AB9 antibodies were obtained in the IgG1 format. The AB10 antibody is a dimer of two chains consisting of VHH fused with the Fc fragment of murine IgG2a.

Figure A1 shows examples of the interaction of antigens’ blood dendritic cell antigen 2 of the cynomolgus macaque (cBDCA-2), poliovirus receptor (NECL-5), triggering receptor expressed on myeloid cells 2 (TREM-2), colony-stimulating factor 1 receptor (CSF1R), and tyrosine-protein kinase receptor UFO isoform 1 precursor (AXL) with antibodies specific to these antigens. The antibodies were immobilized on ProA sensors. A decay of signal was observed during the association stage for AB7–AB10 antibodies.

### Appendix A.1. Empirical Model for Describing Signal Decay During the Association Stage

A model for describing the stage of association of an antigen with antibodies immobilized on Fc receptors or on protein A was obtained with several simplifications. The contributions corresponding to antibody dissociation and accelerated dissociation of antibody–antigen complexes were subtracted from the interaction signal (1:1 model). Firstly, it was assumed that the signal decay caused by the dissociation of antigen–antibody complexes from Fc receptors could be described by mono-exponential dependence. Secondly, it was assumed that all complexes dissociate in an identical manner (regardless of the number of antigens bound to the antibody) and that the proportion of α molecules that have formed a complex with the antigen at fixed time is proportional to the antigen association signal in the standard 1:1 (one to one) interaction model. Thirdly, it was assumed that the signal decay for antibodies not bound to the antigen (their proportion, respectively, is 1 − α) occurred similarly to the reference sensorgram (at the association stage, only the buffer solution was present). Thus, an empirical equation was used to approximate the stage of association of experiments with a sequential measurement scheme, as follows: receptor loading, antibody immobilization, and antigen association (Figure 1, Figure 2, Figure 3 and Figure 4).

(A1) $$I\;=\;R_{\left(1:1\right)}\;-\;\alpha (R_{0}\;-\;I_{\mathrm{diss}})\;-(1\;-\;\alpha )(R_{0}\;-\;I_{\mathrm{ref}})$$

where I is the model signal level at the association stage, R_(1:1)_ is the signal of antigen–antibody binding according to the 1:1 interaction model, described by Equation (A2), α is the proportion of antibodies bound to at least one antigen, described by Equation (A3) (assumption), R_0_ is the initial value of the signal at the association stage, I_diss_ is the value of the decay signal described by Equation (A4), and I_ref_ is the value of the reference curve decay signal at time t (an experimentally obtained set of data values was used).

(A2) $$R_{\left(1:1\right)}\;=R_{0}\;+R_{m}(1\;-\;e^{-t(k_{a}c+k_{\mathrm{diss}})})$$

where R_m_ is the highest achievable signal due to the available number of Fab fragments of antibodies, k_a_ is a constant of the association rate, k_diss_ is a constant of the dissociation rate, c is the concentration of the antigen in the solution, and t is the time counted from the beginning of the association stage.

(A3) $$\alpha =(R_{\left(1:1\right)}\;-\;R_{0})/R_{m}$$

(A4) $$I_{\mathrm{diss}}\;=\;R_{0}\;-\;R_{\mathrm{AB}}(1\;-\;e^{-{\mathrm{tk}}_{\mathrm{model}}})$$

where R_AB_ is a change in the signal level at the stage of antibody immobilization (a signal corresponding to antibodies bound to Fc receptors or protein A), and k_model_ is a constant describing the rate of exponential decay (the functional type of decay is an assumption) of the signal of antibodies bound to one or two antigens (the simplification is that we consider such molecules to be equivalent).

For the approximation, the initial parameters were the values of the constants k_a_ 1.34 × 10^5^ M^−1^s^−1^ and 1.79 × 10^5^ M^−1^s^−1^ for antibodies AB1 and AB2, respectively, k_diss_ 3.72 × 10^−5^ s^−1^ (AB1) and 1.05 × 10^−3^ s^−1^ (AB2) obtained in the standard measurement of kinetics with the immobilization of antibodies on AR2G sensors (Sartorius). The initial R_AB_ value was estimated from the immobilization step on the sensorgrams and varied lastly within small limits if necessary. R_0_ is determined by the beginning of the association stage from sensorgram data (fixed value). I and I_ref_ were the signal values on the sensorgrams of the sample and reference (a discrete set of values for corresponding time values with a step of 0.2 s). The value of R_(1:1)_ was calculated for each corresponding value of t (from 0 s with a step of 0.2 s) according to Equation (A2). The value of α was calculated for each corresponding value of R_(1:1)_ using Equation (A3). I_diss_ were calculated for each time value with a step of 0.2 s (similar to the time step of the values of I, I_ref_) using Equation (A4). The concentration c was specified during the measurements (fixed). To reduce the number of fitting variables, we fixed k_diss_. Fixing this constant could most likely indirectly influence the selected values of the parameters R_m_ and k_a_, so the constants k_a_ and k_diss_ are suitable only for the comparative analysis of the samples within the framework of one measurement method. The values of k_a,_ k_model_, and R_m_ were fitted sequentially until the highest value of the coefficient of determination (R^2^) was reached, then the procedure of sequential fit of k_a,_ k_model_, and R_m_ were repeated several times until it was possible to improve R^2^. The visualization of the model parameters and the approximation process are shown in Figure A2, using the example of the AB2 antibody immobilized on protein A and the NECL-5 antigen.

**Table A1:** Examples of fitted parameter values (Equation (A1)) of an empirical model describing the association with signal decay.

Model Parameters	ProA		FcRn		FcγRIa		FcγRIIIa	
	AB1	AB2	AB1	AB2	AB1	AB2	AB1	AB2
R_0_, (nm)	4.4430	5.7630	1.5720	1.8220	2.8830	3.3140	1.4990	2.0450
R_m_, (nm)	1.105	0.99	0.1	0.36	0.635	0.185	0.128	0.182
R_AB_, (nm)	4.2	5.7	0.4	0.918	2	2.4	2	1.33
k_a_, (M^−1^s^−1^)	1.20 × 10^4^	5.20 × 10^4^	1.80 × 10^5^	2.00 × 10^4^	2.65 × 10^5^	9.90 × 10^4^	4.70 × 10^5^	3.95 × 10^4^
k_diss_, (s^−1^)	3.72 × 10^−5^	1.08 × 10^−3^	3.72 × 10^−5^	1.08 × 10^−3^	3.72 × 10^−5^	1.08 × 10^−3^	3.72 × 10^−5^	1.08 × 10^−3^
k_model_, (s^−1^)	1.00 × 10^−7^	3.28 × 10^−5^	1.61 × 10^−4^	6.00 × 10^−2^	5.50 × 10^−5^	7.44 × 10^−4^	2.05 × 10^−4^	1.90 × 10^−1^
c, (nM)	456.6	284.09	913.2	568.18	456.6	284.09	456.6	284.09
R^2^	0.9048	0.9929	0.9182	0.9984	0.9652	0.9998	0.9562	0.9968

### Appendix A.2. Comparison of Samples by Steady-State Analysis

For experiments on the interaction of antibody–antigen complexes with immobilized Fc receptors, equilibrium state analysis can be applied. Equation (A5) describes the dependence of the equilibrium binding signal (Req) on the concentration of antibodies (or antibody–antigen complexes).

(A5) $$R_{eq}=\frac{cR_{max}}{c+KD}$$

where R_eq_ is the equilibrium binding signal, c is the concentration of the analyte (antibodies or antibody–antigen complexes), R_max_ is the highest achievable signal level due to the available number of receptors on the sensor surface, and KD is the equilibrium dissociation constant. An example of the application of data approximation using Equation (A5) is shown in Figure A4 for the AB2 antibody and AB2 complexes with NECL-5, in relation to immobilized FcγRIIIa. The example demonstrates a decrease in the affinity (increase in KD) of the AB2 antibody to FcγRIIIa by about 1.7 times when the antigen NECL-5 is added. The molar ratio of AB2 to the NECL-5 antigen was 1:0.8; at a molar ratio of 1:2, the signal of the complexes becomes lower than the signal of the antibody by approximately an order of magnitude.
